# Multi-target computational pipeline for discovery of pan-influenza neuraminidase inhibitors

**DOI:** 10.3389/fphar.2026.1721276

**Published:** 2026-03-10

**Authors:** Smbat Gevorgyan, Marusya Ayvazyan, Levon Kharatyan, Anastasiya Shavina, Narek Abelyan, Hamlet Khachatryan, Hovakim Zakaryan

**Affiliations:** 1 Laboratory of Antiviral Drug Discovery, Institute of Molecular Biology, NAS, Yerevan, Armenia; 2 Denovo Sciences Inc., Yerevan, Armenia; 3 Biocentric.ai, Yerevan, Armenia

**Keywords:** broad-spectrum antivirals, cross-MD validation, influenza A/B, molecular dynamics, neuraminidase inhibitors, structure-based drug design, virtual screening

## Abstract

The continuous evolution of influenza A and B viruses, coupled with the emergence of drug resistance, creates a pressing need for novel antiviral agents with broad-spectrum activity. The viral neuraminidase enzyme remains a prime target, but its structural variability across different strains complicates the discovery of universal inhibitors. To address this challenge, we developed and implemented a multi-target computational pipeline designed to identify pan-influenza neuraminidase inhibitors. Our strategy involved high-precision molecular docking of a curated library containing 499,721 compounds against three structurally distinct neuraminidase representatives from influenza A (H1N1, H2N2) and influenza B viruses. Hits were prioritized using a cascade of energetic and geometric filters, followed by a rigorous two-tiered validation using extensive molecular dynamics simulations. This validation not only confirmed binding stability on the primary target but also critically assessed whether candidates maintained stable interactions across the other neuraminidase subtypes. This cross-validation approach was essential for eliminating subtype-specific binders, ultimately identifying ten compounds with robust, pan-influenza binding profiles. Notably, the successful identification of a diastereomer of the established drug zanamivir among the top candidates provides strong validation for the pipeline’s ability to find biologically relevant scaffolds. Overall, this work demonstrates the integration of multi-target screening with cross-validated molecular dynamics (cross-MD) that overcame target variability and yielded ten promising hits candidates for next-generation anti-influenza therapeutics.

## Introduction

Influenza is a persistent global health threat, responsible for an estimated one billion infections, 3–5 million cases of severe illness, and 290–650 thousand respiratory deaths annually. This mortality is likely a significant underestimate, as it omits non-respiratory complications like myocardial infarction and stroke triggered by the infection’s systemic inflammatory nature. The burden is disproportionately borne by vulnerable populations, including older adults, young children, pregnant women, and individuals with chronic medical conditions. Global health inequities magnify this disparity, with 99% of influenza-related deaths in children under five occurring in developing nations where malnutrition and limited healthcare access exacerbate the lethality of the virus (Dharmapalan, 2020; Javanian et al., 2021).

Seasonal influenza is driven by two distinct virus types: influenza A and B. Influenza A, classified into several subtypes such as H1N1 and H3N2, possesses immense genetic diversity and a large zoonotic reservoir, enabling antigenic shifts that can generate novel pandemic strains (Barr et al., 2010; Zambon, 1999). In contrast, influenza B is an almost exclusively human pathogen, divided into the B/Yamagata and B/Victoria lineages, which evolves more slowly through antigenic drift and lacks pandemic potential. While influenza A accounts for approximately 75% of seasonal cases, the perception of influenza B as a milder virus is a misconception. Influenza B can cause severe clinical outcomes, particularly in children, with hospitalization and mortality rates comparable to those of influenza A. This dual threat necessitates the development of pan-influenza inhibitors (Tran et al., 2016; Sharma et al., 2019).

Both influenza A and B rely on the neuraminidase (NA) enzyme for efficient replication. NA is a type II integral membrane glycoprotein that forms a homotetrameric, “mushroom-shaped” spike on the virion surface. In the late stage of the viral life cycle, NA cleaves terminal sialic acid residues from host cell and viral glycoproteins, a crucial step for releasing progeny virions and preventing their aggregation (Air and Laver, 1989; McAuley et al., 2019). Each NA monomer consists of four distinct domains: a short, conserved N-terminal cytoplasmic tail, a hydrophobic transmembrane region that anchors the protein in the viral envelope, a hypervariable stalk region, and a large, C-terminal catalytic head. This globular head folds into a conserved six-bladed β-propeller structure containing the enzymatic active site (McAuley et al., 2019). The assembly of four monomers into a tetramer is an absolute prerequisite for enzymatic function, making both the active site and the inter-subunit interfaces attractive targets for antiviral therapeutics (von Grafenstein et al., 2015).

The highly conserved NA active site contains both catalytic residues (e.g., Arg371), which are essential for cleaving sialic acid, and framework residues (e.g., Glu276), which provide structural integrity (Momont et al., 2023; Yen et al., 2006). This site is the target for cornerstone antiviral drugs known as NA inhibitors, such as oseltamivir and zanamivir, which mimic the sialic acid substrate to halt enzyme activity. While effective, the widespread use of NA inhibitors has driven the emergence of drug-resistant strains carrying mutations (e.g., H274Y, R292K, E119V) that diminish drug binding without compromising viral viability (Batool et al., 2023; Yang et al., 2016). Some circulating influenza A/H1N1 and A/H3N2 isolates have already shown reduced susceptibility to oseltamivir in clinical surveillance. Moreover, zanamivir’s inhaled delivery route and oseltamivir’s moderate effectiveness in severe cases highlight the need for improved therapeutics (Ferraris and Lina, 2008; Lampejo, 2020). In addition, inhibitors act differently on various types of NA, further complicating the situation. The growing threat of NA inhibitor-resistant influenza variants and the limited arsenal of approved antivirals create an urgent need for novel NA inhibitors with broad efficacy and improved resilience against resistance (Świerczyńska et al., 2022).

*In silico* virtual screening has emerged as a powerful strategy to accelerate the discovery of new antiviral agents (Rocha et al., 2019). This structure-based approach utilizes molecular docking to computationally assess the binding affinity of candidate compounds to a target protein, enabling the rapid screening of millions of molecules. It can efficiently prioritize promising leads for experimental testing, vastly reducing the time and cost compared to conventional high-throughput screening (Ferreira et al., 2015; Maia et al., 2020).

In this study, we implement a multi-target virtual screening pipeline to identify novel, broad-spectrum inhibitors targeting three representative NA structures from influenza A (H1N1 and H2N2) and influenza B. Our methodology leverages the structural diversity of multiple NA subtypes by screening a curated 499,721 compound library against each target using the high-precision docking software, ICM-Pro. Initial hits were prioritized through a stringent filtering cascade, combining energetic ranking, visual inspection of binding poses, and geometric constraints to key active site residues. The most promising candidates were then subjected to a rigorous two-tiered validation using molecular dynamics (MD) simulations. This involved first confirming binding stability on their primary target and subsequently performing cross-target simulations to ensure robust binding across different NA structures. Finally, the top ten pan-influenza candidates were tested against the mutant variants of NA and underwent comprehensive ADMET and physico-chemical analysis to evaluate their drug-like properties. This hierarchical strategy, which uniquely integrates multi-target screening with cross-MD, has successfully identified promising compounds with potential as broad-spectrum anti-influenza agents.

## Materials and methods

### Library construction

A comprehensive screening library was assembled from ZINC20 by applying physicochemical and availability filters to enrich for purchasable, drug-like compounds. Molecules were retained if they had molecular weight 200–500 Da, logP ≤ 5, neutral overall charge, and were represented in their predominant form at approximately physiological pH (∼7.0). Only entries labeled “In-Stock” were included to ensure immediate commercial availability. ZINC20 entries contain fully specified stereochemistry; therefore, distinct stereoisomers were treated as separate compounds at this stage. After filtering, compounds were processed in RDKit (v2025.03) and encoded as Morgan fingerprints (radius 2, 1,024 bits). Fingerprints were assembled into a sparse matrix and reduced using Truncated SVD, followed by K-means clustering (k = 1,000) to partition the filtered chemical space into chemically distinct regions. To construct a diverse yet tractable screening set, up to 500 compounds were randomly sampled from each cluster. The choice of 1,000 clusters provided sufficient granularity to separate major chemotypes without fragmenting the space into many sparsely populated clusters, while the 500-compound cap prevented densely populated, highly redundant regions from dominating the final library.

### Target protein structure selection and preparation

Three crystallographic structures of influenza NA were retrieved from the Protein Data Bank based on the following selection criteria: availability in holo form, absence of mutations, chronological recency and the highest resolution. The selected wild-type structures were 6HP0 (Influenza A, H1N1) (Zima et al., 2019), 3TIA (Influenza A, H2N2) (Vavricka et al., 2011), and 4CPY (Influenza B) (Escuret et al., 2014). The mutant structures were 5NWE (N1 neuraminidase H275Y mutant) (Pokorná et al., 2018) and 4GZT (N2 neuraminidase D151G mutant) (Zhu et al., 2012). Each structure was visualized and analyzed using PyMOL (v3.1, Schrödinger, LLC).

Before docking studies, the crystallographic structures were preprocessed by removing water molecules, non-structural ions, and any other non-protein entities present, while the structural calcium ions were retained. The bound ligands were also removed, leaving only the protein coordinates. These cleaned protein structures were then used as the starting point for subsequent docking analyses.

### Virtual screening by ICM

Large-scale virtual screening was performed on a library of 499,721 compounds using Molsoft ICM-Pro software (v3.9) (Abagyan et al., 1994; Abagyan and Totrov, 1994). Before docking, all receptor structures were prepared by removing crystallographic water molecules to optimize the computational efficiency. This step preserved the essential structural features for ligand recognition (Totrov and Abagyan, 1997). The docking calculations employed a docking effort parameter of 10, which represents a ten-fold extension of the default Monte Carlo simulation length to ensure thorough conformational sampling (Bottegoni et al., 2009). For each ligand, up to 15 distinct conformations were generated and evaluated during the docking process to adequately explore the conformational space and identify optimal binding modes (Schapira et al., 1999). The binding site was defined using ICM-Pro’s automated pocket detection algorithm, which constructs a rectangular grid box encompassing all residues within the active site. This built-in function extends the grid boundaries 4 Å beyond the outermost atoms of the surrounding residues, providing sufficient space for ligand accommodation while maintaining computational efficiency (An et al., 2005; Neves et al., 2012). The grid maps were calculated with a standard spacing of 0.5 Å (Totrov and Abagyan, 2008).

Conformational sampling and scoring were performed using ICM’s Biased Probability Monte Carlo BPMC algorithm. This stochastic optimization method combines random conformational moves in internal coordinate space with local energy minimization steps to efficiently explore the energy landscape (Metropolis et al., 1953; Li and Scheraga, 1987). The scoring function incorporated five distinct interaction potentials: van der Waals interactions for both hydrogen and heavy-atom probes, optimized electrostatic terms, hydrophobic contributions, and a lone-pair-based potential accounting for directional hydrogen bonding preferences (Totrov and Abagyan, 2001). All docking calculations were completed by 15 January 2024. The top-scoring poses for each ligand were saved and ranked according to their ICM docking scores for subsequent analysis and validation (Katritch et al., 2010).

### Distance calculation

To evaluate the docking results, a geometric filter was applied to the docking poses. Compounds were selected for further analysis if the minimum Euclidean distance between any of their atoms and the atoms of the catalytic site residues glutamic acid and arginine (Glu277 and Arg368 in 6HP0, Glu276 and Arg371 in 3TIA, Glu274 and Arg373 in 4CPY) was less than 5 Å. A distance of up to 5 Å represents a range within which interactions between atoms may occur. However, mere proximity does not guarantee the formation of an interaction, therefore; the binding conformations of these compounds were then visually inspected using the PyMOL molecular visualization system to assess potential interactions. To obtain an unbiased analysis of ligand interactions with the arginine and glutamic acid residues, the binding poses of the compounds were analyzed using the PLIP (Protein–Ligand Interaction Profiler) (Schake et al., 2025) web tool (v2.3.0). PLIP automatically detects and quantifies specific non-covalent interactions. Based on this analysis, compounds were classified into three categories: (1) compounds predicted to interact with both arginine and glutamic acid residues; (2) compounds predicted to interact with arginine but not glutamic acid; and (3) compounds that were in proximity (<5 Å) but may not interact with either arginine or glutamic acid. No compounds were predicted to interact with glutamic acid in the absence of interactions with arginine. The top compounds were selected for MD simulations by prioritizing those with favorable interaction profiles and low ICM docking scores.

### Molecular dynamics

The MD simulations were performed using the Uni-GBSA tool (v0.1.6) with the GROMACS engine (v2022.4) in MD mode to generate automated trajectories for protein–ligand complexes (Yang et al., 2022). The Amber99SB-ILDN force field was applied to the protein, while ligand topologies were generated with GAFF using AM1-BCC charges through ACPYPE. Complexes were placed in a triclinic simulation box with at least 1.1 nm padding from the solute to the box edge, solvated with TIP3P water, neutralized, and brought to 0.15 M NaCl to mimic physiological ionic strength. Energy minimization was first conducted to remove steric clashes, followed by equilibration under NVT and NPT ensembles for 50,000 steps each, using a 2 fs integration step. Temperature was maintained at 310 K with a velocity-rescale thermostat, while pressure was controlled at 1 bar with the Parrinello–Rahman barostat during equilibration and production runs. All bonds involving hydrogen were constrained using LINCS, and periodic boundary conditions were applied in all directions. Long-range electrostatics were computed using the particle mesh Ewald method with a 1.0 nm real-space cutoff and Fourier grid spacing of approximately 0.12 nm, while van der Waals interactions were treated with a 1.0 nm cutoff and force switching. Each production simulation consisted of 50,000,000 steps, corresponding to 100 ns, and 5,000 trajectory frames were saved at 20 ps intervals. All MD trajectory analyses, including protein Cα and ligand heavy-atom RMSD, the RMSF of the protein Cα (Welford, 1962), hydrogen-bond occupancy, and protein–ligand contact persistence, were performed in Python with MDAnalysis (v2.9) (Michaud-Agrawal et al., 2011). Trajectories were reimaged under periodic boundary conditions and aligned to the protein backbone before analysis. Hydrogen bonds were defined using a donor–acceptor distance cutoff of 3.5 Å and a donor–hydrogen acceptor angle ≥150°.

To assess robustness to resistance-associated substitutions, we additionally simulated the top 10 hit candidates plus oseltamivir for 100 ns using the same MD protocol on two mutant neuraminidase structures: N1 H275Y (PDB 5NWE; corresponding to the commonly cited H274Y depending on numbering) and N2 D151G (PDB 4GZT).

### MM/GBSA

The Molecular Mechanics, General Born Surface Area (MM/GBSA) energies were computed for molecules selected after docking, utilizing 20 frames from short (0.02 ns) molecular dynamics (MD) simulations based on the minimum energy pose produced by docking. The short MD simulations and MM/GBSA calculations were executed using the Uni-GBSA. The default.ini configuration file was employed for both MD simulations and MM/GBSA calculations, utilizing its default parameters, GB calculation mode, and modified MD simulation parameters (nsteps = 10,000, nframe = 20).

### PCA analysis

Principal component analysis (PCA) was performed to characterize both global and binding-pocket motions of the neuraminidase–ligand complex during MD simulations. Trajectories were first aligned to a reference structure using protein backbone (Cα) atoms to remove overall translation and rotation. The covariance matrix of atomic positional fluctuations was computed from the aligned trajectories, and its eigenvectors and eigenvalues were extracted, representing collective motions and their corresponding amplitudes, respectively. For the whole-protein PCA, backbone atoms were used to construct the covariance matrix and analyze the dominant global motions. For the binding-pocket PCA, residues within 5 Å of the ligand in the initial frame were identified and only their Cα atoms were included to isolate local fluctuations. In both analyses, the first principal components—capturing the majority of the total variance—were examined, and cumulative variance plots as well as PC1 vs. PC2 projections were generated to visualize the major conformational transitions of the system. To characterize protein dynamics, residue-wise contributions were calculated using a subset of principal components (PCs) accounting for 60% of the cumulative variance. For each PC, the Euclidean norms of the eigenvector components were first normalized to sum to unity across all residues. These standardized values were then weighted by the normalized variance of their respective PC and summed to determine the final contribution of each residue to the collective motion. Highly flexible residues were identified as those with a contribution score at least one standard deviation above the median. To minimize noise from the N-terminus, only residues beyond index 30 were considered for this selection. All PCA analyses were performed using MDAnalysis.

### Physicochemical and ADMET analysis

For the selected compounds, key physicochemical properties were calculated, including: molecular weight (MW), topological polar surface area (TPSA), number of hydrogen bond acceptors (HBA) and donors (HBD), octanol-water partition coefficient (logP), rotatable bond count, ring count, and the fraction of sp^3^-hybridized carbon atoms (Fsp^3^). These properties were compared with known NA inhibitors exhibiting sub-10 µM activity, as retrieved from PubChem and ChEMBL bioassay data (Kharatyan et al., 2025). We calculated each molecule’s mean Tanimoto similarity coefficient against the inhibitor set to assess structural similarity between the ten selected compounds and known NA inhibitors. The analysis employed Morgan fingerprints (radius = 2, nBits = 2048) as the molecular representation method. The ADMET (Absorption, Distribution, Metabolism, Excretion, and Toxicity) properties of the ten candidate compounds were predicted using the ADMET-AI platform (Swanson et al., 2023) and compared against known NA inhibitors.

### PDB interaction analysis

To analyze the observed distributions of interacting amino acids in the discussed proteins, we retrieved all deposited protein structures resolved by X-ray crystallography with 100% sequence identity and a resolution better than 2.5 Å for all investigated neuraminidase subtypes. Overall, 9 structures were found and retrieved based on the above-mentioned criteria, based on the 6HP0 reference structure. Consequently, interacting amino acids were identified and analyzed using the PLIP toolkit.

### Data analysis and visualization

Data analysis, visualization and interatomic distance calculation were performed in Python (v3.10, NumPy, SciPy, Matplotlib, Seaborn, Biopandas). Cheminformatics tasks (standardization, property calculation, fingerprints, and 2D depictions) used RDKit; tabulation and aggregation used pandas. Final figure assembly and editing were done in Inkscape (v1.4) and Figma (v125.4).

## Results

### Library construction

The cluster-balanced sampling strategy improved chemical space representation while preserving computational tractability for high-precision docking. Partitioning the filtered pool into 1,000 clusters and applying a 500-compound cap per cluster limited the influence of highly populated analogue-rich regions and reduced redundancy in the final screening library. In a size-matched comparison (499,721 compounds), the cluster-sampled library showed higher diversity than a purely random subset from the same starting pool, as quantified by a lower mean Tanimoto similarity (0.1294; lower indicates higher diversity). Random sampling produced a higher mean Tanimoto similarity, consistent with preferential selection of dominant scaffolds in the source database. Together, these results support that the selected parameters yield a more balanced and diverse starting library for subsequent virtual screening.

### Virtual screening and hit selection

**FIGURE 1:**
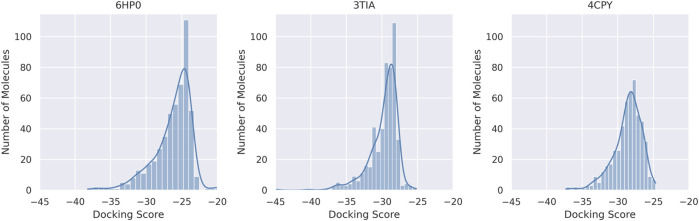
The distributions of ICM docking scores for the top 500 compounds against the three targets (6HP0, 3TIA, and 4CPY).

The virtual screening campaign of the compounds was performed against three distinct protein targets (PDB IDs: 6HP0, 4CPY, and 3TIA) to identify promising hit candidates based on their predicted binding affinities. For every compound-protein combination, the best-scoring pose was identified from the 15 conformations generated during the docking simulation. From this initial library, the top 500 compounds were selected for each of the three targets based on their ICM docking scores. The ICM docking scores distributions for the top 500 compounds selected for each target revealed a broad range of binding energies, from ICM scores −44.9 to −19.4 (Figure 1). Within this set, only one compound was common to all three targets, only one compound overlapped between 6HP0 and 3TIA NA structures, two compounds overlapped between 6HP0 and 4CPY, and 35 compounds were overlapped between 3TIA and 4CPY. For 6HP0, the compound ZINC000100425730 exhibited the best ICM docking score (−38.2). The best score for 3TIA was observed for ZINC000014768526 (−44.9) and for 4CPY it was ZINC000257330770 (−37.3). Oseltamivir was used as a reference compound, with ICM docking scores of −24.9, −39.2 and −32.6 for 6HP0, 3TIA, and 4CPY, respectively.

### Distance analysis

**FIGURE 2:**
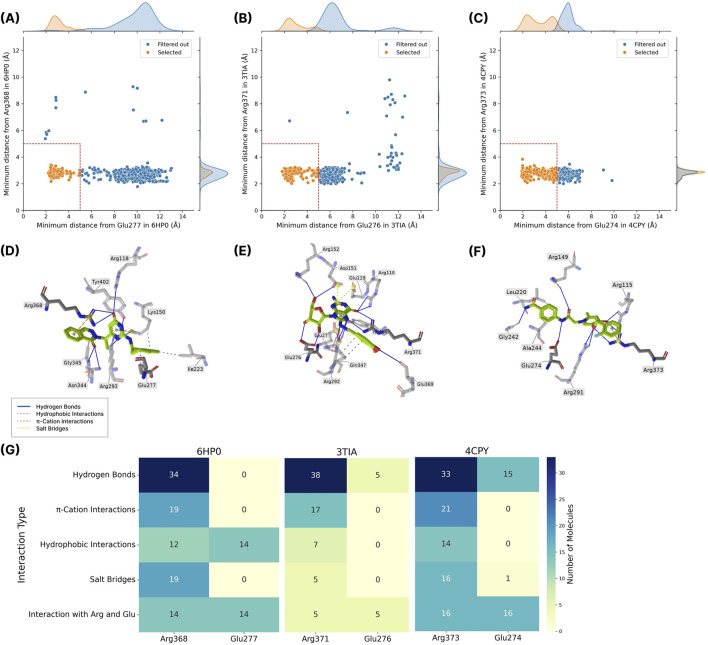
**(A–C)** Panels show the minimum distances of docked compounds from the key residues Glu and Arg across three protein structures: **(A)** Glu277 and Arg368 in 6HP0, **(B)** Glu276 and Arg371 in 3TIA, and **(C)** Glu274 and Arg373 in 4CPY. Each point represents a single compound. Compounds classified as *Selected* (orange) if both distances are below 5 Å, and *Filtered out* (blue) if distances are higher from one or both amino acids. The red dashed lines mark the cutoff threshold. Kernel density estimates are displayed along the axes to illustrate the distribution of distances. **(D–F)** Panels display representative 3D visualizations of a “Selected” compound bound within the active site of each respective protein target. Key non-covalent interactions are highlighted: hydrogen bonds (blue lines), hydrophobic interactions (grey dashed lines), π–cation interactions (orange dashed lines), and salt bridges (yellow dashed lines). **(G)** The heatmap presents the types and frequencies of interactions between the compounds selected for MD simulations and the key residues.

To further filter the top hit candidates, we analyzed their binding poses within the catalytic active site of NA. We focused on Glu276 and Arg371 in 3TIA (corresponding to Glu277 and Arg368 in 6HP0, and Glu274 and Arg373 in 4CPY), which are key residues that directly interact with sialic acids (McAuley et al., 2019). Both residues have been shown to participate in interactions with various ligands, as evidenced by the analysis of available holo structures of NA (Supplementary Figure S1A). Moreover, these two amino acids are positioned on opposite sides (outer and inner) of the catalytic site (Supplementary Figure S1B), hence, if a compound is rationally designed to be close to both of the amino acids, it will naturally occupy the whole binding pocket. Therefore, we measured the distances between the top hit candidates and these two key amino acid residues and prioritized those ligands that exhibited a distance of less than 5 Å from both residues, hypothesizing that the compounds geometrically fill the catalytic pocket and may form favorable interactions. The ICM scores and distance values of the top 500 candidates are listed in Supplementary Table S1. Figures 2A–C shows the minimum distance values of the compounds relative to key amino acids. A total of 102 compounds for 6HP0, 135 compounds for 3TIA and 272 compounds for 4CPY exhibited a minimum distance of less than 5 Å from both residues.

Then, we conducted visual inspection and PLIP analysis to select compounds that may form interactions with the mentioned amino acid residues. The highest priority was given to compounds predicted to interact with both amino acids, followed by those predicted to interact only with Arg371 in 3TIA (Arg368 in 6HP0 and Arg373 in 4CPY). Compounds showing no predicted interactions were excluded from further analysis. For compounds exhibiting similar interaction profiles, priority was given to those with lower docking scores. Figures 2D–F shows 3D visualizations of representative examples of selected compounds and their interactions with 6HP0, 3TIA, and 4CPY, respectively.

Based on visual inspection, PLIP and the docking energies, a total of 37, 40, and 40 compounds were selected for MD simulations with 6HP0, 3TIA, and 4CPY, respectively. PLIP analysis of these compounds indicated that, across all protein structures, a greater number of compounds interact with Arg than with Glu, with hydrogen bonding being the most common type of interaction. In 3TIA and 4CPY, the compounds mainly interact with glutamic acid through hydrogen bonding, whereas contacts to Glu277 in 6HP0 were predominantly non-hydrogen bonds (Figure 2G).

### MD simulations

**FIGURE 3:**
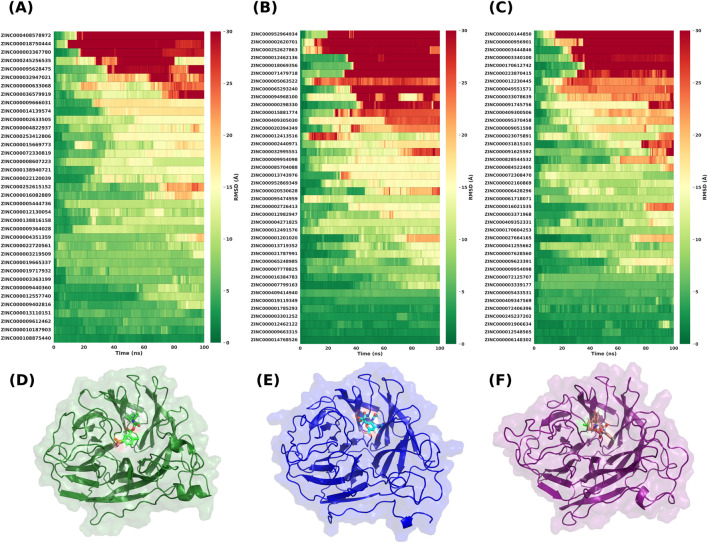
**(A–C)** Ligand stability heatmaps for neuraminidase targets 6HP0 (n = 37), 3TIA (n = 40), and 4CPY (n = 40) based on 100-ns explicit-solvent MD simulations in GROMACS using Uni-GBSA. Trajectory frames were superposed on protein Cα atoms. Each row represents a ligand (ZINC ID shown on the y-axis), and the x-axis indicates simulation time in nanoseconds. Colors represent ligand heavy-atom RMSD in Å, with scale bars at right (0–30 Å; green indicates low, red indicates high RMSD). Ligands within each panel are ordered by their time-averaged RMSD over the 0–100 ns trajectory. **(D–F)** Representative bound-state snapshots for the same targets: 6HP0, 3TIA, and 4CPY, respectively. Proteins are shown as ribbons with a semitransparent surface; ligands are depicted as sticks within the active site.

We advanced 117 top ligands to explicit-solvent MD: 37 for 6HP0, 40 for 3TIA, and 40 for 4CPY. Each protein–ligand complex was simulated for 100 ns in GROMACS under the conditions described in the Methods section. Figures 3A–C summarizes ligand stability with time-resolved ligand RMSD heatmaps and representative bound-state snapshots. Ligand stability was assessed by ligand heavy-atom RMSD after superposition of trajectory frames on protein Cα atoms. Within each target, ligands were ranked by their time-averaged RMSD across 0–100 ns. Low, sustained RMSD indicates retention in the active site with limited pose drift; high RMSD indicates pocket escape or large translational/rotational drift. As a practical guide, values ≤3–5 Å denote tight pocket retention, whereas values ≥15–20 Å are consistent with partial or complete egress. Across all three structures a consistent pattern emerged. The absence of systematic drift and the observation of plateau-like behavior in the RMSD trajectories served as confirmation that the systems reached a stable equilibration regime suitable for assessing relative stability.

A subset of compounds maintained uniformly low RMSD throughout the trajectory, while another subset showed rapid escalation to >15–20 Å within the first 20–40 ns, indicating loss of the binding mode or exit from the pocket. For 6HP0, ZINC000108875440 displayed the lowest RMSD value, consistently remaining below 3 Å across the trajectory, whereas ZINC000408578972 drifted to high RMSD values. For 3TIA, ZINC000014768526 was the most stable ligand, remaining below ∼3 Å for essentially the entire 100 ns, while ZINC000952964934 exhibited persistently high RMSD. For 4CPY, ZINC000245237202 was the most stable, while ZINC000020144850 ranked worst by RMSD due to early and sustained excursions. We compared the 3D structures in their holo forms of the three crystallographic targets (Figures 3D–F). Visual inspection reveals that, despite superposition, the structures exhibit noticeable differences in conformation and shape. Overall, these MD analyses result in a set of candidates with stable pocket retention for subsequent computational studies including cross-subtype MD simulations, hydrogen-bond analysis, and free energy calculation via MM/GBSA.

### Cross-MD simulations

To test whether the selected top hit candidates form stable complexes with all types of NA, we performed cross-MD simulations: ligands that were stable on one NA structure were re-simulated for 100 ns on the other two NA structures. From the primary MD screens, we took the top 13 ligands discovered on 6HP0 and simulated them on 3TIA and 4CPY; the top 11 discovered on 3TIA and simulated them on 6HP0 and 4CPY; and the top 11 discovered on 4CPY and simulated them on 6HP0 and 3TIA. Trajectories were aligned to the protein backbone, and ligand heavy-atom RMSD was tracked over time with respect to the bound pose used to start the simulation. The overall results for all cross-validated ligands revealed clear heterogeneity across the systems (Supplementary Figure S2). While several ligands retained compact, flat RMSD trajectories on the alternative structures—consistent with a preserved binding mode—others displayed rapid RMSD increases within the first 20–40 ns followed by a plateau at high values, indicating pocket exit or major rearrangement. For decision-making, we summarized each trajectory by the mean RMSD and high-percentile behavior and defined “pan-NA stability” as sustained low RMSD without persistent late-trajectory spikes on all three backbones. Ten ligands met this criterion and were advanced for further analysis: ZINC000014768526, ZINC000108875440, ZINC000245237202, ZINC000019119349, ZINC000091906634, ZINC000019717932, ZINC000012462122, ZINC000072125707, ZINC000003339177, and ZINC000001785293 (Table 1).

**TABLE 1:** Structure, chemical class, mean RMSD and MM/GBSA values of the top 10 compounds compared to the oseltamivir control.

Compound ID	Structure	Chemical class	MM/GBSA (kcal/mol)	Mean MD RMSD (Å)
ZINC000014768526	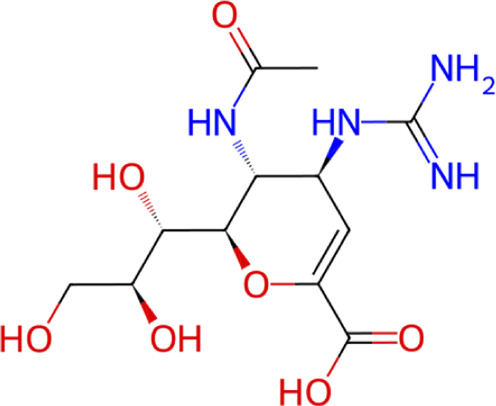	Sialic acid analog, dihydropyran derivative	−69.7	2.37
ZINC000108875440	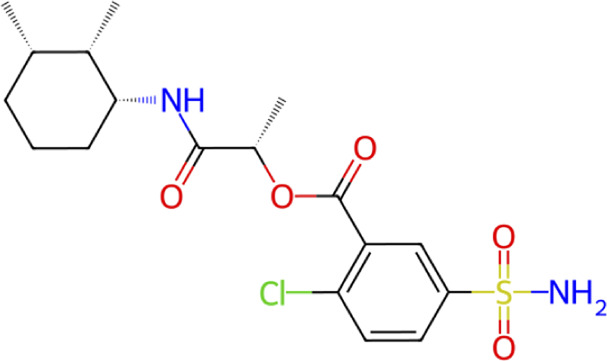	2-chloro-5-sulfamoylbenzoic acid derivative	−52.2	3.21
ZINC000245237202	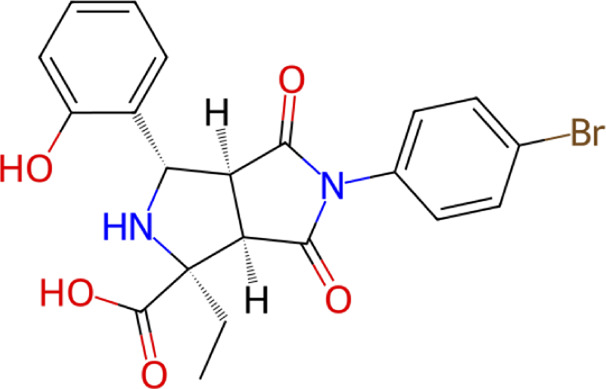	tetrahydropyrrolo[3,4-c]pyrrole-1,3-dione derivative	−58.9	3.53
ZINC000019119349	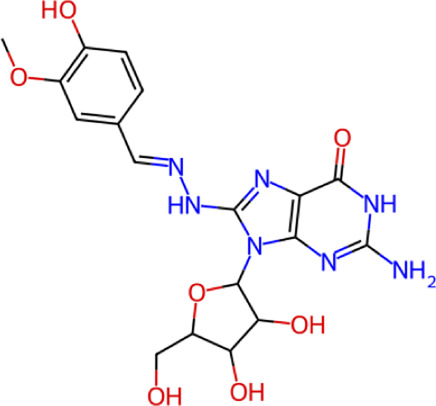	Purine nucleoside derivative	−40.5	3.77
ZINC000091906634	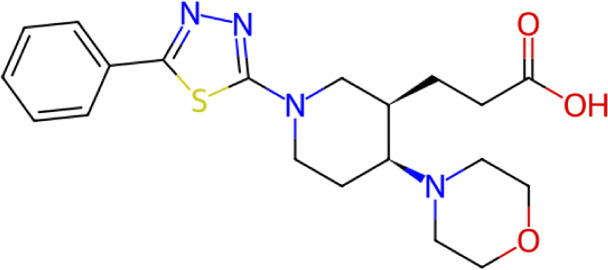	1,3,4-thiadiazole piperidine derivative	−66.2	4.00
ZINC000019717932	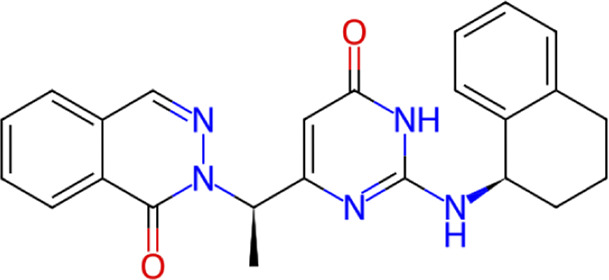	Phthalazinone pyrimidine derivative	−40.5	5.14
ZINC000012462122	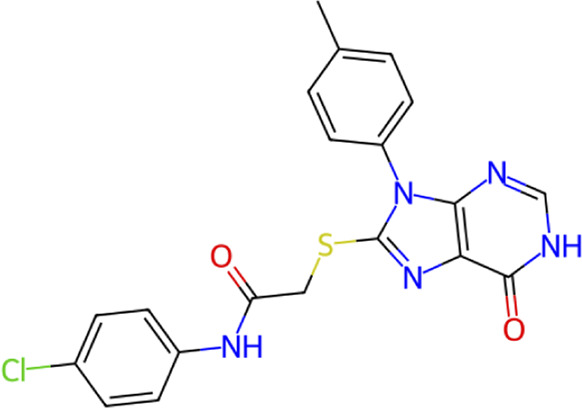	Purin-6-one thioacetamide	−30.4	5.68
ZINC000072125707	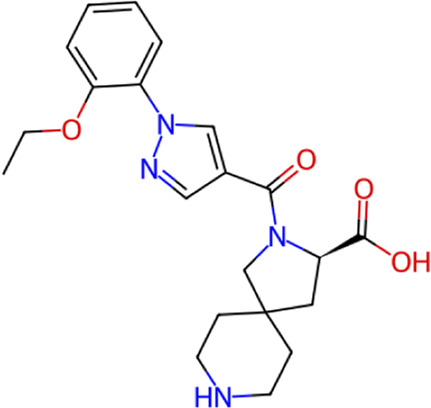	Spirocyclic pyrazole acid	−63.8	5.86
ZINC000003339177	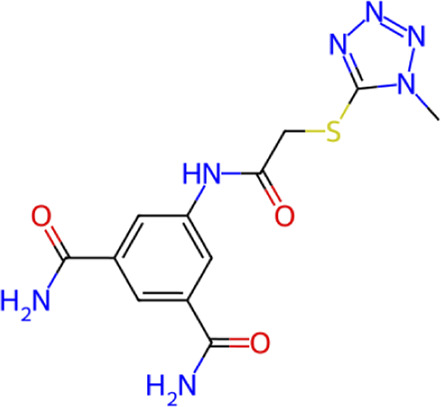	Tetrazole-isophthalamide	−44.9	6.30
ZINC000001785293	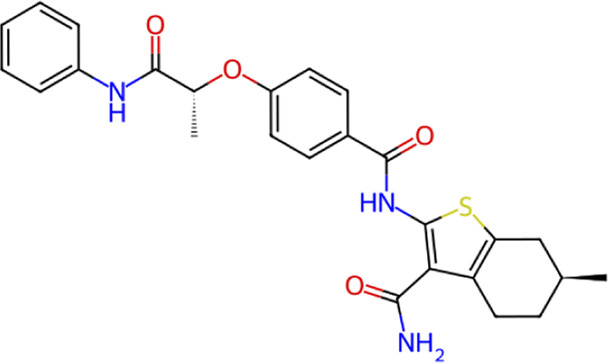	Tetrahydrobenzothiophene derivative	−45.4	6.31
Oseltamivir	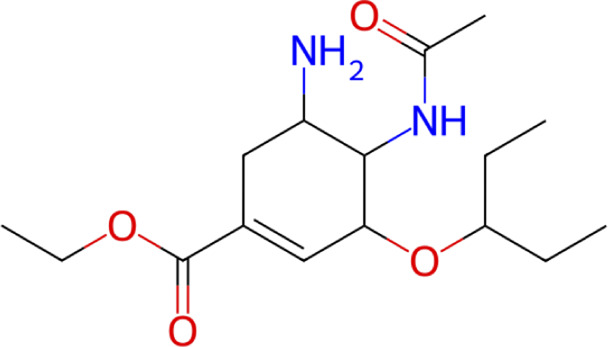	Sialic acid analog	−50.3	4.22

**FIGURE 4:**
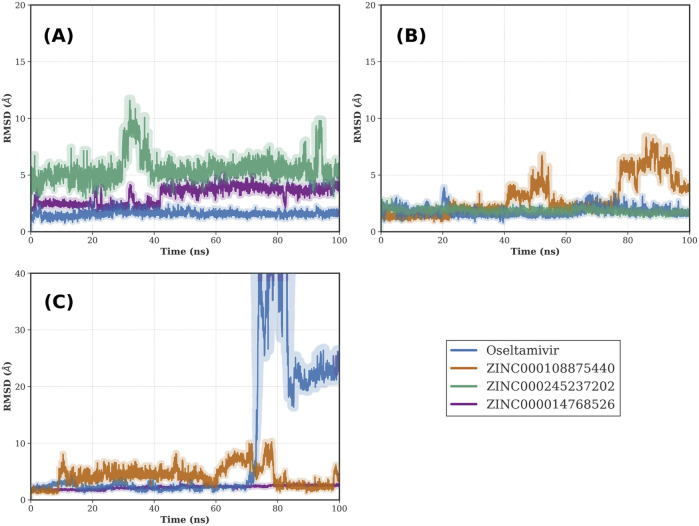
Comparative cross-MD stability of the top three pan-NA candidates versus Oseltamivir. The plots display ligand heavy-atom RMSD trajectories over 100 ns MD simulations for the three most promising inhibitors (ZINC000014768526, ZINC000108875440, and ZINC000245237202) and the reference drug oseltamivir docked into three distinct neuraminidase structures: **(A)** 6HP0 (Influenza A/H1N1), **(B)** 3TIA (Influenza A/H2N2), and **(C)** 4CPY (Influenza B). Solid lines represent the mean RMSD values derived from independent simulation replicas, while the shaded bands indicate the corresponding standard deviations, demonstrating the high reproducibility of the top hits.

Among these, three compounds, ZINC000014768526, ZINC000108875440, and ZINC000245237202, demonstrated the most robust and consistent stability, yielding the lowest mean RMSD values across the three structures. To visualize this superior stability and rule out stochastic artifacts, we performed independent replication of the MD simulations. The mean RMSD trajectories and standard deviations for these top three candidates, alongside the reference drug oseltamivir, are plotted in Figure 4.

Oseltamivir demonstrated remarkable stability on the influenza A structures (6HP0 and 3TIA), with RMSD values remaining below 2 Å throughout the simulation. However, on the influenza B structure (4CPY), oseltamivir exhibited significant instability; from approximately 70 ns onward, the RMSD rose sharply, reaching above 40 Å, indicating ligand displacement. In contrast, the top three candidates maintained compact, stable profiles across all three viral subtypes, characterized by narrow standard deviation bands (Figure 4), lower mean RMSDs, and more favorable MM/GBSA binding free energies than the control (Table 1). Notably, each of these top performers originated from a batch screened on a different primary structure (3TIA, 6HP0, and 4CPY, respectively), arguing against structure-specific overfitting and supporting true cross-subtype robustness.

The top-ranked candidate, ZINC000014768526, showed exceptional reproducibility, maintaining stable RMSD values in the replica runs across all three protein structures. Similarly, the replicas for ZINC000108875440 and ZINC000245237202 confirmed the binding modes observed in the initial screen. This validation confirms that the reported stability represents a robust energetic minimum rather than a transient sampling event.

Structural analysis of the top-ranked candidates revealed that ZINC000014768526 is a diastereomer of the known drug zanamivir (Supplementary Figure S3), an identity that was not recognized prior to the MD analysis. This finding provides a strong independent validation for our computational pipeline; a close structural analog of an approved drug not only passed the entire screening cascade but also emerged as the top-ranked candidate based on its predicted metrics. A retrospective analysis was then performed on the entire curated library of 499,721 compounds (Supplementary Table S2) to identify any other isomers of known NA targeting drugs. This search confirmed that the zanamivir diastereomer was the sole such compound present, and it was the only one to successfully navigate the complete workflow.

Taken together, the cross-MD results validate that a subset of candidates preserves a stable binding mode across diverse NA backbones, thus excluding ligands that are sensitive to backbone-specific features. The ten stable molecules were prioritized for deeper analysis, including hydrogen bond analysis, physicochemical and ADMET studies.

### Mutant stability assessment

**FIGURE 5:**
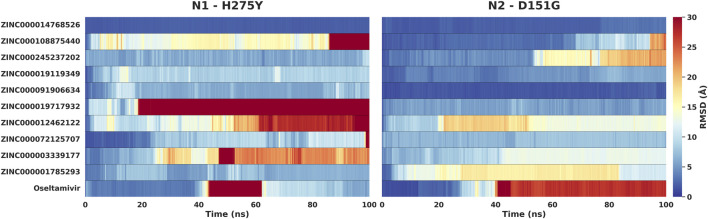
MD stability of top candidates against clinically resistant neuraminidase mutants. The heatmaps display the time-averaged ligand RMSD (Å) for the top 10 candidates and oseltamivir over 100 ns simulations in the active sites of the A/H1N1 H275Y mutant (PDB ID: 5NWE) and the A/H3N2 D151G mutant (PDB ID: 4GZT). Blue indicates high stability (low RMSD), while red indicates high instability or ligand dissociation. Note the distinct failure of oseltamivir compared to the robustness of ZINC000014768526.

Given that the clinical efficacy of NA inhibitors is frequently compromised by resistance-associated mutations, the wild-type and cross-subtype MD validations were complemented with a targeted mutant stability assessment. Specifically, the top 10 compounds alongside oseltamivir, were subjected to 100 ns MD simulations bound to two experimentally determined mutant NA structures: the H275Y mutant of the N1 subtype (PDB: 5NWE) and the D151G mutant of the N2 subtype (PDB: 4GZT). Ligand stability was quantified using heavy-atom RMSD relative to the initial bound conformation and summarized in RMSD heatmaps (Figure 5), enabling a direct, compound-by-compound comparison across mutant contexts.

In the N1 H275Y system, several hit candidates (e.g., ZINC000014768526, ZINC000245237202, ZINC000019119349, ZINC000072125707, and ZINC000001785293) maintained persistently low RMSD values throughout the trajectory, indicating tolerance to this clinically significant substitution. Conversely, a subset of compounds displayed clear destabilization, characterized by partial or sustained displacement from the binding pocket. This included ZINC000019717932 (early transition to high RMSD) and ZINC000012462122 (progressive drift culminating in high RMSD), while ZINC000108875440 demonstrated late-trajectory instability. Oseltamivir exhibited significant dynamic instability, characterized by a spontaneous dissociation from the binding pocket mid-trajectory. Although the ligand subsequently re-entered the active site towards the end of the simulation (indicated by the return to lower RMSD values), this transient unbinding event highlights the weakened affinity and kinetic instability imposed by the H275Y mutation. These distinct behavioral patterns confirm that the H275Y mutation imposes a significant structural perturbation, successfully differentiating robust versus mutation-sensitive chemotypes within the lead set.

Within the N2 D151G structure, the majority of compounds remained stable or exhibited only moderate positional drift without sustained high-RMSD behavior. Notably, ZINC000014768526 demonstrated exceptional stability across both mutant structures, highlighting its potential as a broad-spectrum NA inhibitor. In contrast, oseltamivir displayed pronounced destabilization, exiting the binding pocket at approximately 40 ns and remaining displaced for the duration of the simulation. Collectively, this mutant MD assessment identifies a subset of hit candidates that preserve binding stability not only across NA subtypes but also under resistance-associated perturbations. This provides computational evidence that our multi-target NA pipeline can discover hit candidates with robust binding properties to both native and mutant NAs.

### PCA analysis

**FIGURE 6:**
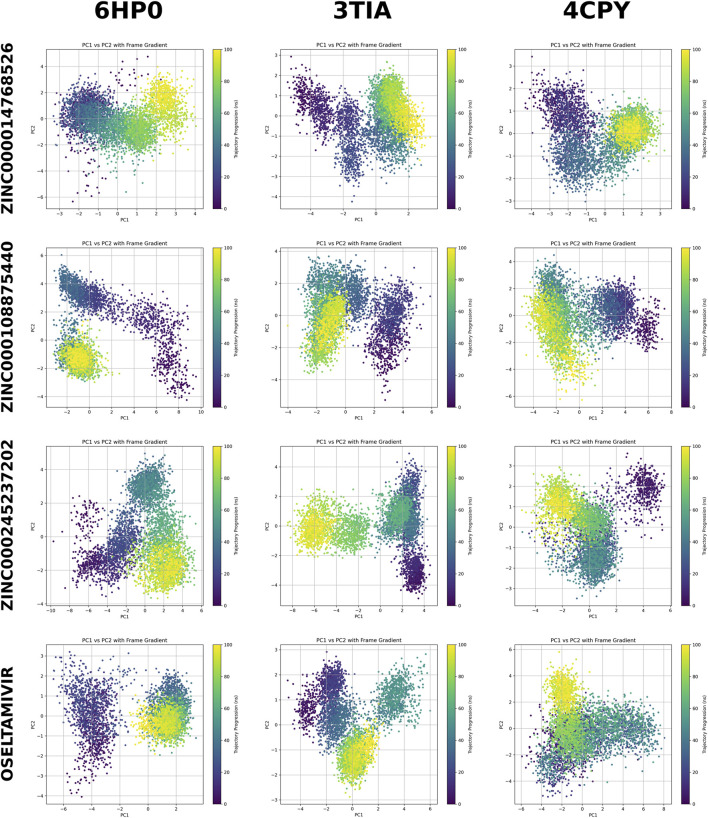
Principal component analysis (PCA) of binding-pocket residues for neuraminidase subtypes 6HP0, 3TIA, and 4CPY in complex with the top three screened ligands (ZINC000014768526, ZINC000108875440, ZINC000245237202) and oseltamivir. Each scatter plot shows PC1 vs. PC2 colored by simulation time (frame gradient), illustrating the conformational pathways sampled during the 100 ns simulations.

PCA of both the binding-pocket residues (Figure 6) and the entire neuraminidase proteins (6HP0, 3TIA, 4CPY) (Supplementary Figure S4) in complex with the top three ligands (ZINC000014768526, ZINC000108875440, ZINC000245237202) and the reference inhibitor oseltamivir revealed that, although trajectories explored different regions of conformational space over time, the final frames of most complexes consistently converged into a well-defined, stable region, as reflected by clustering in the corresponding PCA projections. In the binding-pocket analysis, PC1 captured the dominant motions of the active site, while PC2 reflected more localized rearrangements. Most ligand complexes exhibited two dominant conformational populations, although several systems showed additional smaller clusters depending on the ligand and neuraminidase subtype.

**FIGURE 7:**
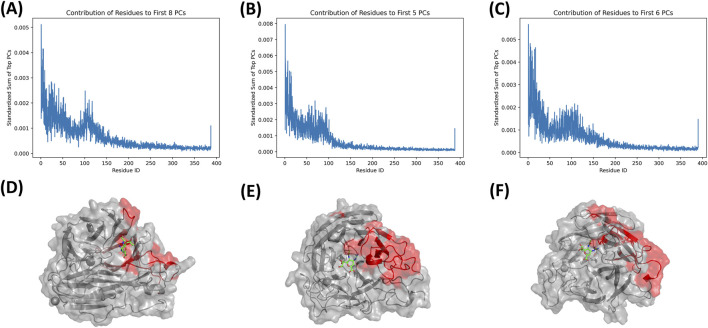
Residue-wise contributions to essential dynamics for three neuraminidase complexes with the zanamivir diastereomer. Line plots **(A–C)** show the standardized per-residue contribution summed over the leading principal components required to reach 60% cumulative variance for each system (6HP0, first 8 PCs; 3TIA, first 5 PCs; 4CPY, first 6 PCs). Structural renderings **(D–F)** map the highest-contributing regions onto the corresponding neuraminidase structures, with flexible hotspots highlighted in red and the ligand shown as green sticks. In all complexes, elevated contributions are observed at the N- and C-termini, along with distinct internal peaks indicating localized conformational flexibility. In 3TIA, prominent hotspots localize to the pocket-gating loop and residues forming the binding-pocket walls, consistent with opening and closing motions of the active site. In 6HP0, major contributions cluster near the same loop and adjacent pocket-shaping elements. In contrast, 4CPY displays dominant flexible regions largely distal to the binding pocket, indicating a different global dynamic profile. Panels A and D correspond to 6HP0, panels B and E to 3TIA, and panels C and F to 4CPY.

In contrast, whole-protein PCA demonstrated broader and more globally coordinated motions. Although PC1 and PC2 still separated the systems into clear dynamical states, the conformational clusters were more elongated and continuous compared to the pocket-restricted PCA space. Across all ligands, 3TIA sampled the widest protein-level conformational space, whereas 6HP0 showed the most compact distribution, indicating a more structurally rigid framework. 4CPY maintained an intermediate profile. Ligand-dependent effects were also observable at the whole-protein level, although they were less pronounced than in the pocket-only analysis. Across all four ligands, the temporal color gradients indicated that, despite detectable conformational shifts during the simulations, most complexes ultimately converged to well-defined final clusters in both the pocket and whole-protein PCA spaces, supporting stable and converged sampling of the conformational landscape. Quantitatively, PC1 captured the majority of the essential dynamics, accounting for 19.0%–43.8% of the global protein variance (Supplementary Table S3) and 23.9%–57.8% of the binding-pocket variance (Supplementary Table S4) across the studied complexes. Figure 7 illustrates the residue-wise contributions to the essential dynamics (derived from the principal components accounting for 60% of the cumulative variance) for different neuraminidases in complex with zanamivir diastereomer. In all complexes, high flexibility is observed at the N- and C-termini. Between these terminal regions, several distinct peaks emerge, representing internal zones of high conformational flexibility. In the 3TIA complex, these flexible regions include the loop, which is responsible for the opening and closing of the binding pocket, as well as residues constituting the pocket walls. Similarly, in 6HP0, flexible residues are located near this loop and contribute to the binding pocket architecture. Conversely, in 4CPY, the majority of the highly flexible residues are located distal to the binding pocket, suggesting a different dynamic profile for this complex.

These PCA-derived essential dynamics were supported by residue-level flexibility (RMSF) profiles computed for all three structures in complex with the top 10 compounds and oseltamivir (Supplementary Figures S5–S7). Across all three neuraminidase backbones, RMSF values remained below 3 Å for most residues, while key active-site residues showed RMSF values below 1 Å, indicating that the protein folds remained stable throughout the simulations. In Supplementary Figures S5–S7, binding-pocket residues are highlighted as red points and key residues are indicated by arrows. Residue indices shown on the x-axes correspond to the renumbered GROMACS topology, which differs from PDB numbering due to missing residues in the crystallographic structures.

### Hydrogen bond analysis

**FIGURE 8:**
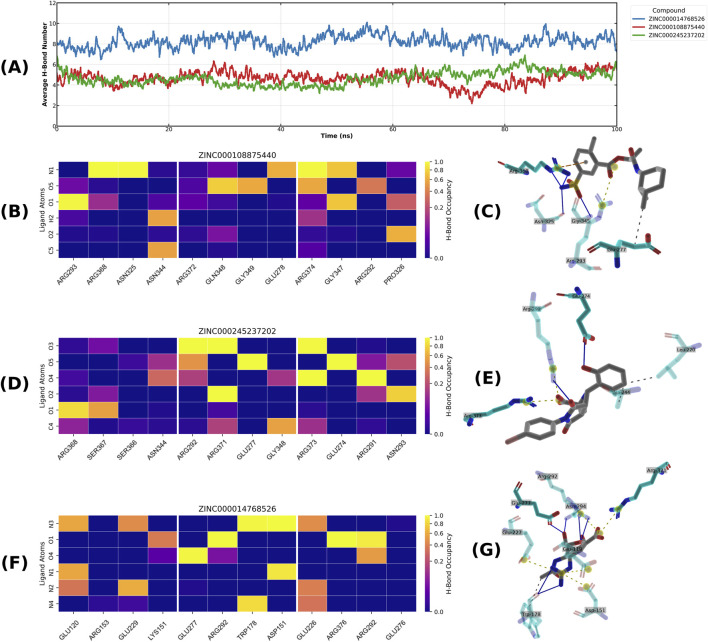
Hydrogen bond analysis of top pan-neuraminidase inhibitor candidates. **(A)** Average number of hydrogen bonds maintained by the top three candidate compounds (ZINC000108875440, ZINC000245237202, and ZINC000014768526) with their averages for all three NA targets over the 100 ns MD simulations, with 0.5 ns rolling mean. **(B,D,F)** Hydrogen bond occupancy heatmaps for the top three compounds across the three target structures (6HP0, 3TIA, and 4CPY). The color scale represents the percentage of simulation time a specific hydrogen bond is maintained, from 0% (dark blue) to 100% (yellow), highlighting dynamically persistent interactions. **(C,E,G)** 2D interaction diagrams of the lowest energy binding poses from the MD simulations. Dashed gray lines are hydrophobic interactions, solid blue lines - hydrogen bonds, dashed yellow lines - salt bridges, dashed orange lines - π-cation Interactions, yellow circles - charge centers. Atom colors: blue - nitrogen, red - oxygen, yellow - sulfur, brown - bromine. The specific compounds are **(B,C)** ZINC000108875440, **(D,E)** ZINC000245237202, and **(F,G)** ZINC000014768526.

To elucidate the structural basis for the stability of the top-performing compounds, a detailed hydrogen bond analysis was conducted on the three most consistent pan-neuraminidase binders. The average number of hydrogen bonds maintained throughout the 100 ns simulations is summarized in Figure 8A, highlighting the stable interactions formed by ZINC000108875440, ZINC000245237202, and ZINC000014768526. For ZINC000014768526, the number was around 8 throughout the simulations and for ZINC000108875440 and ZINC000245237202 the mean number of bonds fluctuated around 4–6. The hydrogen bond occupancy heatmap for compound ZINC000108875440 (Figure 8B) revealed that the compound maintained persistent interactions throughout the simulation, with notable high-occupancy bonds involving residues Arg368, Glu277, and Asn325 with 6HP0. These interactions are also visible in the lowest energy pose, as depicted in the 2D interaction map (Figure 8C). This static view highlights a foundational network of hydrogen bonds with these key catalytic residues. In the simulation with the 4CPY structure, compound ZINC000245237202 also demonstrated a robust and stable interaction network. The occupancy heatmap in Figure 8D shows durable hydrogen bonds primarily with residues Arg373, Glu274, and Arg291. These stable contacts over the simulation’s duration are also illustrated by the interactions visualized in the 2D diagram of the lowest energy pose (Figure 8E). This core interaction network likely facilitates the sustained, high-occupancy hydrogen bonds identified in the heatmap, collectively ensuring the compound’s stable residence in the binding pocket. Finally, ZINC000014768526, when analyzed with the 3TIA structure, exhibited the most extensive and stable hydrogen bonding profile of the top candidates. Its heatmap in Figure 8F indicates consistent, high-occupancy bonds with a broad range of active site residues, including Glu276, Arg292, Trp178 and Asp151. The structural basis for this exceptional stability is clearly depicted in the 2D map (Figure 8G), which shows the ligand forming multiple simultaneous hydrogen bonds with the above-mentioned amino acids in addition to multiple other contacts such as with Arg371 and Glu119. This dense network of interactions with key conserved residues provides a clear rationale for its superior stability with not only in 3TIA but across all tested neuraminidase subtypes, further confirming the promising results acquired from MD.

In addition, we calculated the average number of hydrogen bonds across the MD simulations for the remaining seven compounds in the top 10 (Supplementary Figure S8). These compounds formed fewer hydrogen bonds than the top three, with substantial variability. For example, ZINC000001785293 and ZINC000019717932 showed very few hydrogen bonds, whereas ZINC000019119349 and ZINC000003339177 consistently formed at least two to three hydrogen bonds throughout their MD trajectories.

Finally, by comparing the binding free energy values, hydrogen bonding interactions and RMSD stability of the top 10 compounds, we observed that, the top-ranked compound, ZINC000014768526, also exhibits the most favorable MM/GBSA energy (−69.7 kcal/mol), which in turn correlates strongly with its exceptional stability (lowest mean RMSD of 2.37 Å) and its extensive and persistent hydrogen bond network (maintaining an average of ∼8 H-bonds). The other two pan-NA binders, ZINC000108875440 and ZINC000245237202, show similarly coherent behavior, combining favorable MM/GBSA estimates (−52.2 and −58.9 kcal/mol) with low mean RMSD values (3.21 and 3.53 Å) and sustaining ∼4–6 hydrogen bonds throughout the simulations. Among the remaining seven candidates, ligands with moderately favorable MM/GBSA values and intermediate mean RMSD, such as ZINC000019119349 and ZINC000003339177 (−40.5 and −44.9 kcal/mol; 3.77 and 6.30 Å), consistently form at least two to three hydrogen bonds. In contrast, compounds with weaker MM/GBSA energies and higher RMSD values, including ZINC000019717932 and ZINC000001785293 (−40.5 and −45.4 kcal/mol; 5.14 and 6.31 Å), show very few hydrogen bonds. ZINC000091906634 and ZINC000072125707 illustrate intermediate behavior: both have strong MM/GBSA estimates (−66.2 and −63.8 kcal/mol) but higher mean RMSD (4.00 and 5.86 Å, respectively) and less dense hydrogen-bond networks than the top three, suggesting that they remain bound while sampling multiple orientations. Overall, the MM/GBSA, RMSD, and hydrogen-bond profiles are broadly consistent, with the most favorable binding energies generally associated with more stable trajectories and richer hydrogen-bonding patterns.

### Physicochemical properties

We compared NA-targeting active molecules (activity <10 µM) extracted from PubChem and ChEMBL bioassays with the top ten selected compounds. First, we calculated the mean Tanimoto similarities between the ten selected compounds and the known active molecules (Supplementary Table S5). Only ZINC000014768526, a diastereomer of zanamivir, exhibited a higher mean similarity than the overall diversity (0.239) of the known active molecules.

**FIGURE 9:**
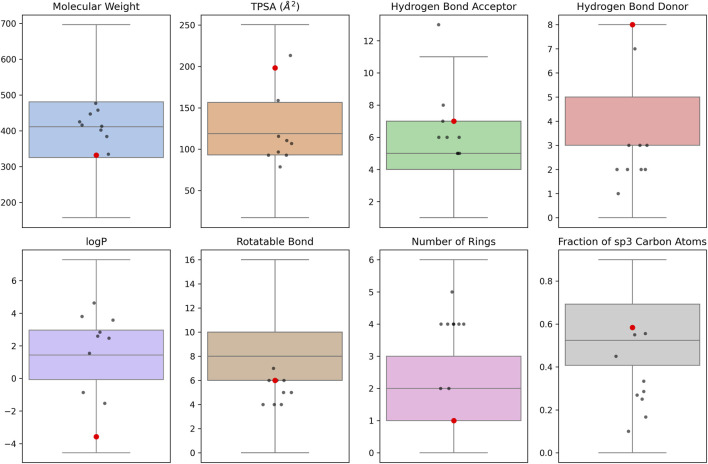
Visualization of key physicochemical and structural properties of ten selected molecules in comparison with NA-targeting active molecules. The distribution of each property among NA-targeting active molecules is shown as boxplots. Black points represent the nine candidate compounds, while the red point denotes the diastereomer of zanamivir.

To enable comparison, we calculated the physicochemical and structural properties of the ten candidate NA inhibitors (Supplementary Table S6) and the known NA-targeting active compounds. For MW, all ten selected molecules fall within the interquartile range of the known NA-targeting molecules, which is 325–480 g/mol (Figure 9). The majority of the selected molecules fall within the central half of the dataset. However, some molecules, including the zanamivir diastereomer, exhibit a TPSA greater than 200 Å^2^, well above the dataset median. This indicates higher polarity, consistent with the presence of numerous hydrophilic functional groups in these molecules. Both hydrogen bond donors and acceptors are critical for NA binding. The diastereomer of zanamivir shows a high donor count (8), standing out as an outlier, and a relatively high acceptor count (∼7). Consequently, this compound violates Lipinski’s Rule of 5 (HBD > 5); however, this is consistent with approved inhibitors like zanamivir and reflects the highly polar nature of the NA active site, which often necessitates properties outside standard drug-like space. Most of the selected molecules fall outside the logP interquartile range of 0–3, which reflects the mild hydrophobic character of the known active molecules. Three of the selected compounds display higher logP values, indicating they are more hydrophobic than the majority of known molecules. In contrast, four molecules, including the ZINC000014768526, exhibit lower logP values. The dataset exhibits a median of eight rotatable bonds and two rings. Most of the selected molecules fall outside the interquartile ranges of these structural properties. The majority possess four or more rings. ZINC000014768526 lies close to the dataset medians. Despite having a high number of rings, the molecules contain fewer rotatable bonds and lower Fsp^3^ values, which is consistent with their structures, as they are composed of non-aliphatic heterocycles.

### ADMET predictions

The interaction probabilities of the ten selected NA-targeting compounds with major cytochrome P450 (CYP450) isoforms were evaluated, including CYP1A2, CYP2C19, CYP2C9, CYP2D6, and CYP3A4 (Supplementary Figure S9). Low interaction probabilities (<0.2) suggest a minimal likelihood of enzyme inhibition, whereas higher values (>0.5) indicate potential drug–drug interaction risks. Most compounds showed low probabilities of CYP inhibition, with values generally below 0.2 across the five isoforms, indicating a favorable safety profile with respect to CYP-mediated metabolism. However, a subset of molecules displayed higher interaction probabilities, highlighting potential liabilities. Specifically, ZINC000108875440, ZINC000019717932, ZINC000001785293, and ZINC000012462122 interact with at least one CYP isoform, with ZINC000012462122 showing interactions with nearly all of them.

**FIGURE 10:**
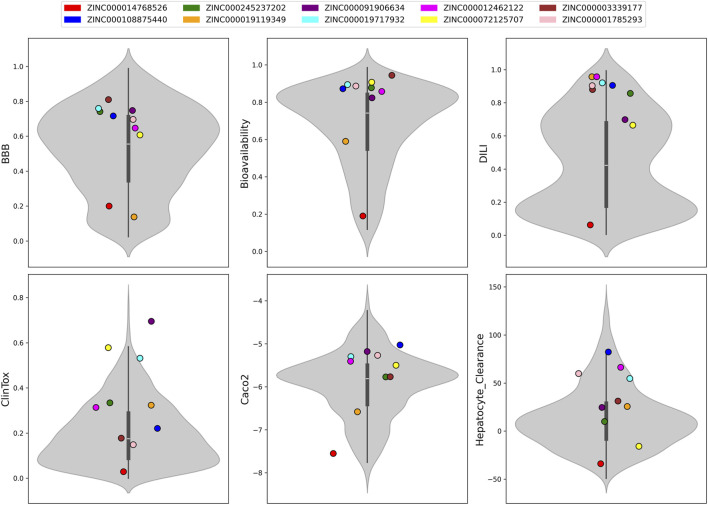
Comparison of additional ADMET properties of the 10 candidate NA inhibitors with those of known NA-targeting active molecules. Distributions for the reference set are shown as violin plots. Colored points indicate individual candidate molecules, with the red point corresponding to the zanamivir diastereomer. The axes BBB, Bioavailability, DILI, and ClinTox represent the probabilities that a compound can cross the blood–brain barrier, be orally bioavailable, induce drug-induced liver injury, and exhibit overall clinical toxicity, respectively. Caco-2 permeability is expressed in cm/s on a logarithmic scale, with higher values indicating greater membrane permeability. Hepatocyte clearance is reported in mL/min/kg, where higher values reflect increased metabolic clearance by hepatocytes.

Some other ADMET properties of the ten candidate NA inhibitors (Supplementary Table S7) were compared with the distribution of known NA-targeting active molecules (Figure 10). Individual candidates are shown as colored points, with the red point representing the zanamivir diastereomer (ZINC000014768526). Most of the selected molecules cluster within the higher range of blood–brain barrier (BBB) penetration probabilities (0.6–0.8). In contrast, the zanamivir diastereomer is positioned closer to the lower range (0.2), indicating poor BBB permeability, which is consistent with the structural properties of zanamivir. The majority of candidates exhibit high bioavailability scores (>0.8), indicating potential for oral administration, and fall within the interquartile range (0.55–0.84). However, the zanamivir diastereomer is positioned at the lower tail (0.19). Most compounds show high drug-induced liver injury (DILI) scores (>0.7), suggesting an elevated risk of hepatotoxicity. While the predicted low BBB permeability is advantageous for minimizing CNS side effects, the elevated DILI risk and low bioavailability suggest that, similar to zanamivir, these compounds may require formulation optimization or alternative delivery routes, such as inhalation, to ensure clinical viability. In contrast, the zanamivir diastereomer again lies at the extreme low end, indicating minimal predicted risk. Conversely, the candidate compounds are broadly distributed across the ClinTox spectrum (μ = 0.34 ± 0.21), while the zanamivir diastereomer is positioned near the lowest range, reflecting low predicted systemic toxicity, consistent with its established clinical safety profile. The interquartile range of Caco-2 permeability for these ten molecules spans from −5.76 to −5.27, indicative of moderate-to-low intestinal absorption. Hepatocyte clearance values show considerable variability across candidates, with a mean of 30.53 mL/min/kg and a standard deviation of 36.82 mL/min/kg. ZINC000014768526 lies at the lowest end of the distribution, reflecting limited hepatic clearance, consistent with zanamivir’s primary renal excretion pathway observed in clinical use (Weller et al., 2013).

## Discussion

The application of computer-aided drug design, particularly structure-based virtual screening and MD simulations, represents a powerful strategy for accelerating the discovery of novel influenza NA inhibitors. Prior *in silico* efforts have spanned classical docking screens, pharmacophore modeling, and focused virtual campaigns on various NA subtypes. Structure-based docking of large libraries has yielded new NA inhibitors (Hsu et al., 2017), but these studies often relied on a single crystal structure per target. Such one-structure approaches risk overfitting to a particular conformation and ignore the enzyme’s flexibility and NA variability across the strains. Ligand-based pharmacophore models have also been employed, for example, models derived from known NA inhibitors that achieved high specificity in distinguishing actives. These pharmacophore screens and traditional docking campaigns, however, tended to be subtype-specific (e.g., targeting H5N1/H1N1 NA only) and did not address cross-strain variation or resistance mutations. Indeed, resistance-associated NA mutations like H274Y in H1N1 and I222R in H5N1 have driven the need for alternative inhibitors (Batool et al., 2016), yet many virtual screens did not explicitly consider such variants, leading to hits that could be rendered ineffective by single amino acid changes. Recently, we demonstrated that our reinforcement learning–based small-molecule design platform is capable of generating compounds that effectively target both native and mutant NAs, with potent antiviral activity confirmed *in vitro* and *in vivo* (Matevosyan et al., 2023; Izmailyan et al., 2024). These works highlight that incorporating multiple NA structures from the outset of computational drug discovery campaigns can be an effective strategy for identifying broadly active compounds, independent of NA subtype.

The present study introduces several strategies to consider the differences in NA structures. First, compounds were docked against multiple NA structures spanning influenza A group-1, group-2, and influenza B NAs. A geometric filtering step then prioritized candidates based on their proximity to highly conserved catalytic amino acid residues in the NA active site. By requiring interactions with key catalytic amino acids such as Glu276 and Arg371, the method focuses on chemotypes likely to bind across subtypes. Finally, explicit-solvent MD simulations were integrated both as a primary screening filter and for cross-target validation. Rather than treating the protein as rigid, the pipeline used MD to refine docked poses and ensure stability in solvent. This dynamic step is leveraged not only on the initial NA target but also to evaluate whether top hit candidates remain stably bound in other NA subtype models: a direct assessment of cross-target transferability that goes beyond the static docking employed in many other studies (Hussain Basha and Prasad, 2012; Mtambo and Kumalo, 2022; Lotfi et al., 2024). Overall, these steps help prevent the selection of hit candidates that only fit a distinct NA structure.

Through our multi-target screening pipeline, we identified hit candidates that formed stable complexes with all three types of NA, suggesting that these compounds may exert potent antiviral activity against influenza viruses regardless of strain. Several top compounds identified in the initial MD simulations were excluded from the final candidate list because they failed to maintain stable complexes with other NA structures. This finding highlights that false-positive results yielded from MD simulations performed on a single NA protein can be minimized by employing cross-MD simulations, as demonstrated in our study. Complementing the ligand stability data, the RMSF analysis confirms that the protein backbones remain structurally rigid upon binding, particularly within the active site where minimal fluctuations (<1 Å) indicate that the candidates are accommodated without inducing destabilizing conformational changes. PCA of both the binding-pocket and whole-neuraminidase proteins revealed that local and global motions are subtype- and ligand-dependent. Binding-pocket PCA identified two main conformational states for most complexes, while whole-protein PCA captured broader, coordinated motions, with 3TIA being the most flexible and 6HP0 the most rigid. Importantly, despite transient conformational shifts during the simulations, most systems converged to a well-defined final state, consistent with clustering patterns observed across both PCA and other MD analyses.

Consistent with these observations, the MM/GBSA, RMSD, and hydrogen-bond analyses converge to highlight the same subset of compounds as the most promising pan-NA inhibitors. Ligands that maintain multiple stable contacts with conserved active-site residues tend to exhibit more favorable calculated binding free energies, whereas compounds with weaker MM/GBSA values also show less stable MD behavior. In this study, we therefore interpret MM/GBSA results qualitatively and in conjunction with the MD-based stability metrics, using their agreement to prioritize candidates with both strong predicted affinity and robust cross-subtype engagement.

Interestingly, the compound ZINC000014768526, which consistently formed highly stable complexes with all three NAs in MD simulations, was identified as a diastereomer of zanamivir. Since our screening library does not include other sialic acid-like NA inhibitors, including zanamivir itself, the emergence of a zanamivir diastereomer among the final hit candidates that successfully passed all filters, including cross-MD validation, provides strong support that our multi-target pipeline is capable of enriching true positives and identifying biologically relevant scaffolds. Beyond cross-subtype breadth, the targeted mutant MD assessment highlights the potential of these leads to overcome established drug resistance mechanisms. The H275Y mutation, the primary driver of oseltamivir resistance in N1 subtypes, compromises drug efficacy by introducing a bulky tyrosine side chain that sterically clashes with the hydrophobic pentyl ether group of the inhibitor. Our simulations successfully recapitulated this phenotype with oseltamivir exhibiting pocket exit and instability. In sharp contrast, the top-performing candidates, most notably ZINC000014768526, maintained robust stability against both the H275Y and D151G mutations. This resilience likely stems from the binding mode identified in our hydrogen-bond analysis. Whereas oseltamivir relies on hydrophobic packing that is vulnerable to mutation, ZINC000014768526 engages the active site through a dense network of polar interactions with conserved residues. By targeting the immutable electrostatic core of the enzyme rather than variable hydrophobic sub-pockets, these “resistance-resilient” chemotypes offer a distinct advantage in evading the rapid evolutionary escape common to influenza viruses.

The distinct instability of oseltamivir observed in our Influenza B (4CPY) simulations mirrors the reduced clinical efficacy of this drug against B-lineage viruses compared to Influenza A strains (Aoki et al., 2007; Sharma et al., 2019). While oseltamivir binds tightly to the N1 and N2 subtypes, structural studies indicate that the Influenza B active site accommodates the inhibitor’s bulky hydrophobic pentyl side chain less effectively due to subtle differences in pocket topology and residue flexibility (Smith et al., 2002; McKimm-Breschkin, 2013). Our MD results, which capture the spontaneous displacement of oseltamivir from the 4CPY pocket, provide a dynamic structural rationalization for these clinical limitations. This finding reinforces the necessity of prioritizing scaffolds like the zanamivir diastereomer, which utilize conserved polar interactions rather than variable hydrophobic contacts to achieve true broad-spectrum retention. While our 100 ns MD simulations successfully validated the immediate binding stability of the top candidates, we acknowledge that this timescale is inherently limited and may not capture rare events such as long-term ligand dissociation. Therefore, future validation studies may be applied to investigate these slower unbinding kinetics through extended microsecond-scale simulations.

Beyond molecular docking and MD simulations, our multi-target screening pipeline integrates ADMET property predictions for the top-ranked hit candidates. Incorporation of ADMET profiling at this stage enhances the reliability of hit selection by excluding compounds with unfavorable pharmacokinetic or toxicological characteristics, such as poor permeability or potential toxicity, thereby reducing the likelihood of attrition in subsequent stages of research (Adelusi et al., 2022). Most of the final hit candidates, including the zanamivir diastereomer, exhibited low predicted probabilities of inhibition across the five major CYP isoforms (CYP1A2, CYP2C19, CYP2C9, CYP2D6, and CYP3A4), suggesting a favorable safety profile with respect to CYP-mediated metabolism. In addition, ADMET predictions suggested a generally low probability of overall clinical toxicity for most compounds. The observed violations of Lipinski’s rules and low oral bioavailability are anticipated for analogs of polar substrates like sialic acid. Since zanamivir itself requires inhaled administration due to these same properties, our candidates would likely follow a similar delivery route, rendering their low oral bioavailability acceptable. Finally, the elevated DILI risk predicted for several compounds serves as an important flag to guide specific toxicity reduction efforts during future lead optimization.

In conclusion, our study demonstrates that a multi-target screening strategy, integrating docking, explicit-solvent primary and cross-MD simulations, and ADMET profiling, provides a robust framework for discovering broad-spectrum NA inhibitors. By testing candidate compounds across influenza A group-1, group-2, and influenza B NAs and retaining only those that maintained stable binding with all NAs, this approach minimizes false positives and enriches true biologically relevant scaffolds such as the zanamivir diastereomer. Our results underscore the importance of incorporating multiple NA structures early in computational drug discovery to identify candidates capable of overcoming NA subtype variability, thereby advancing the development of potent antiviral agents. However, this study represents the initial computational stage of a broader discovery pipeline. To translate these *in silico* findings into clinical solutions, future *in vitro* and *in vivo* testing is required to experimentally validate the biological activity of the identified leads, thereby advancing the development of potent antiviral agents.

## Data Availability

The original contributions presented in the study are publicly available. The library construction data can be found here: https://doi.org/10.6084/m9.figshare.31423367.v1.
