# Supplementary figures and images for: Multi-target computational pipeline for discovery of pan-influenza neuraminidase inhibitors
Source: Front Pharmacol. 2026 Mar 10;17:1721276. doi: 10.3389/fphar.2026.1721276 (PMC13008930; doi:10.3389/fphar.2026.1721276)

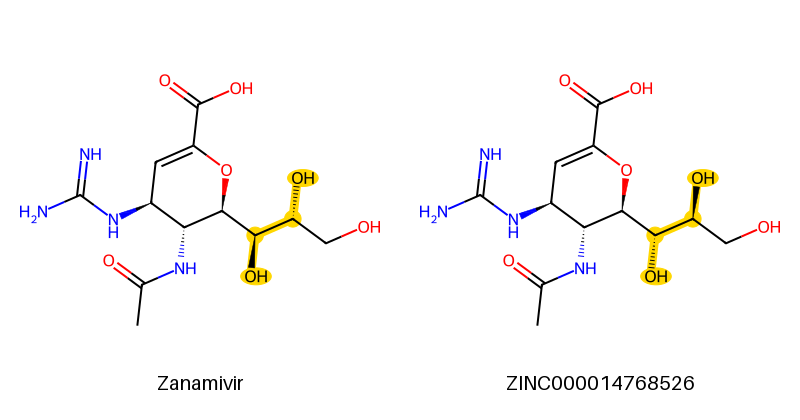

Supplement: Supplementary file 1 [file Image3.tiff]

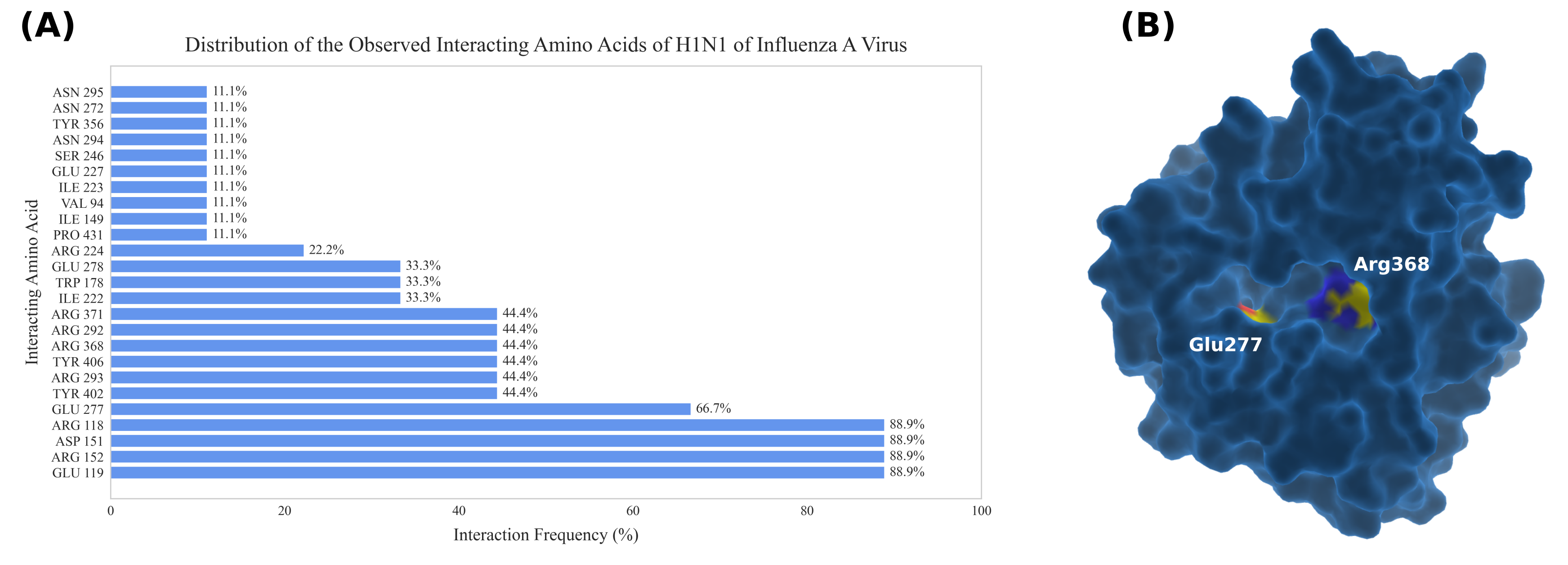

Supplement: Supplementary file 3 [file Image1.tiff]

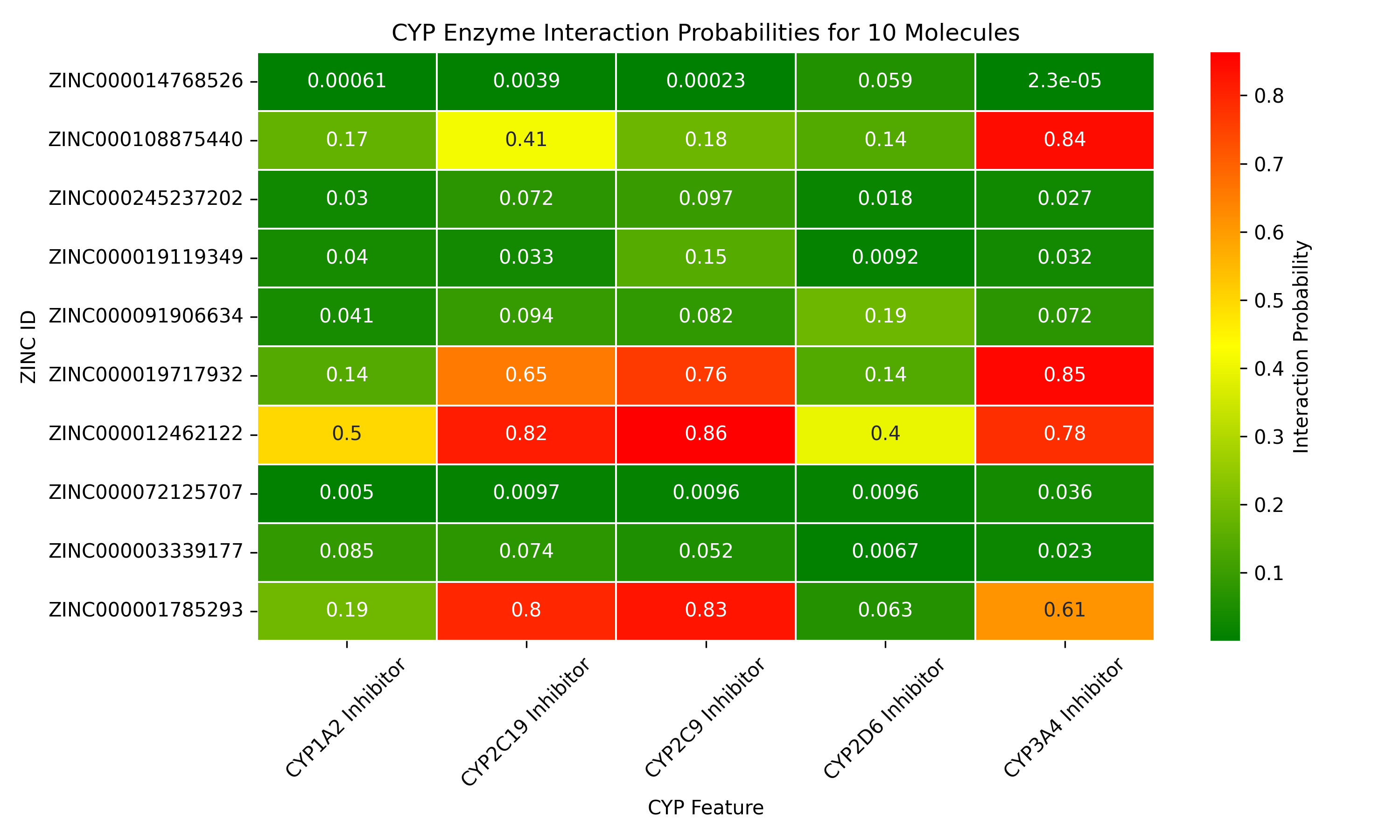

Supplement: Supplementary file 4 [file Image9.tiff]

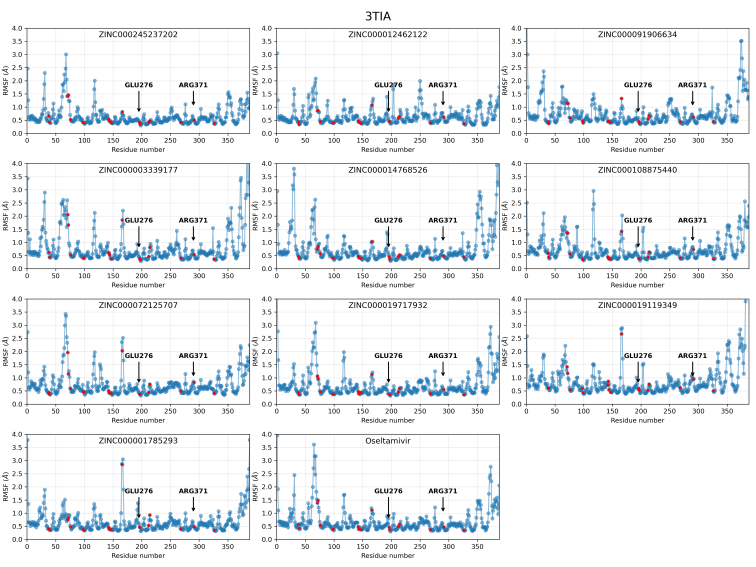

Supplement: Supplementary file 6 [file Image5.png]

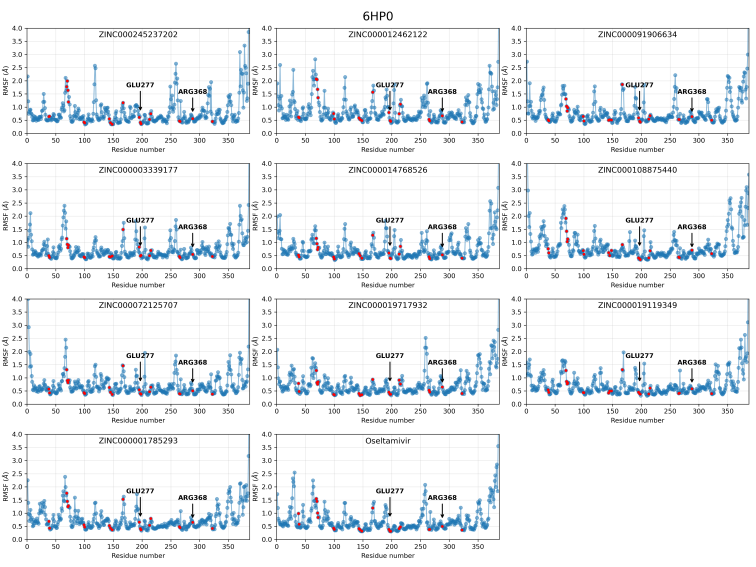

Supplement: Supplementary file 9 [file Image7.png]

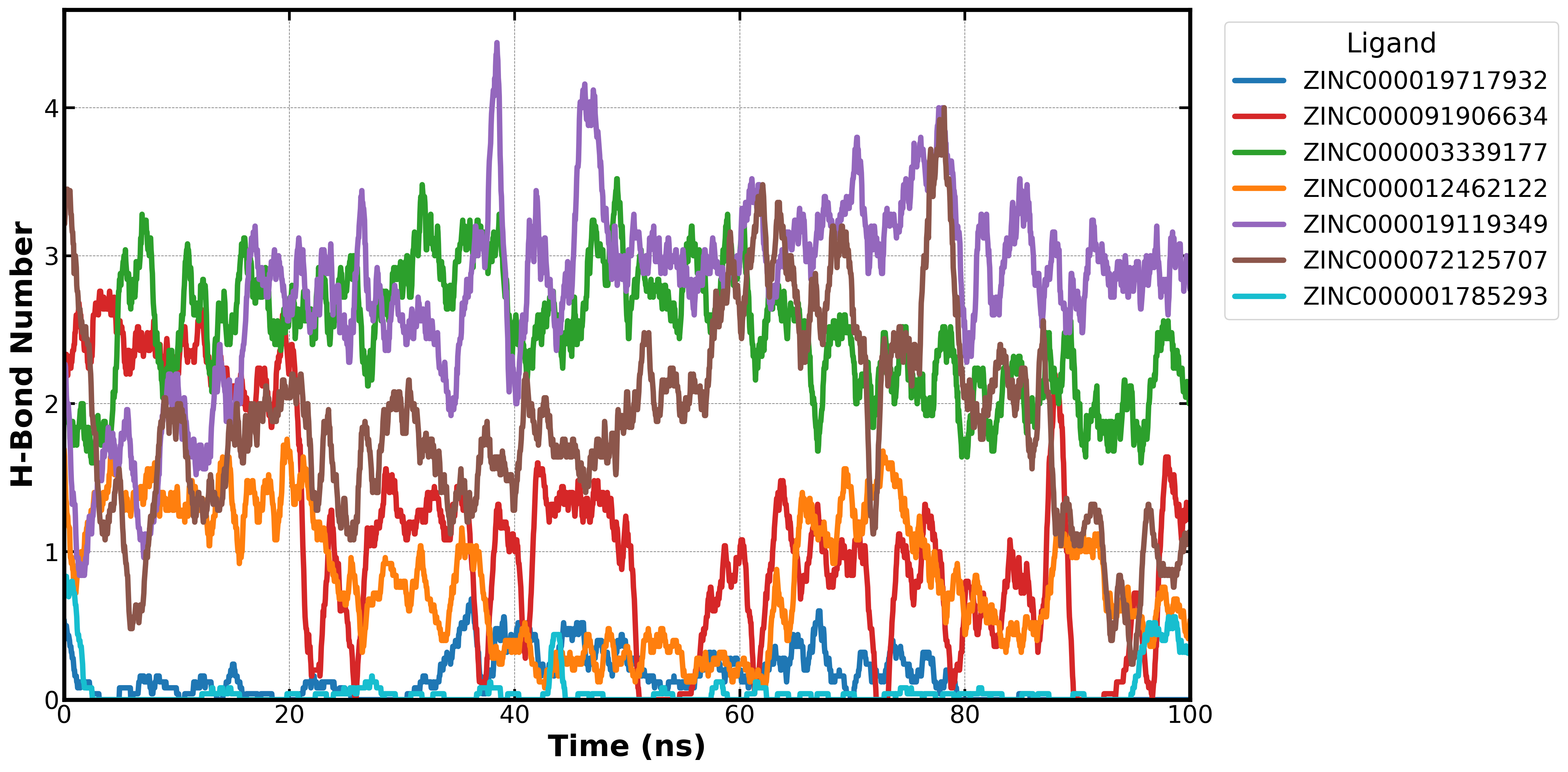

Supplement: Supplementary file 11 [file Image8.png]

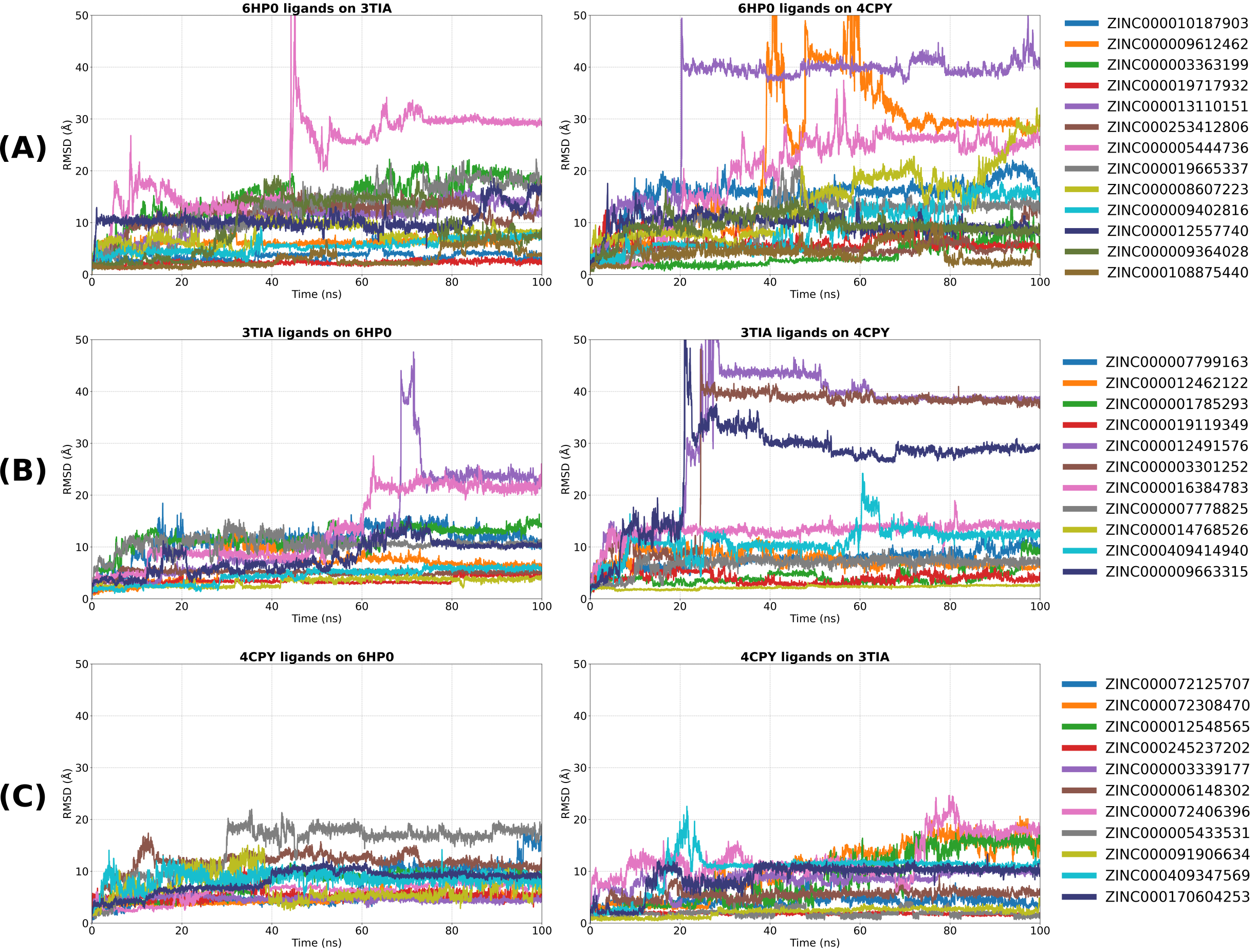

Supplement: Supplementary file 13 [file Image2.tiff]

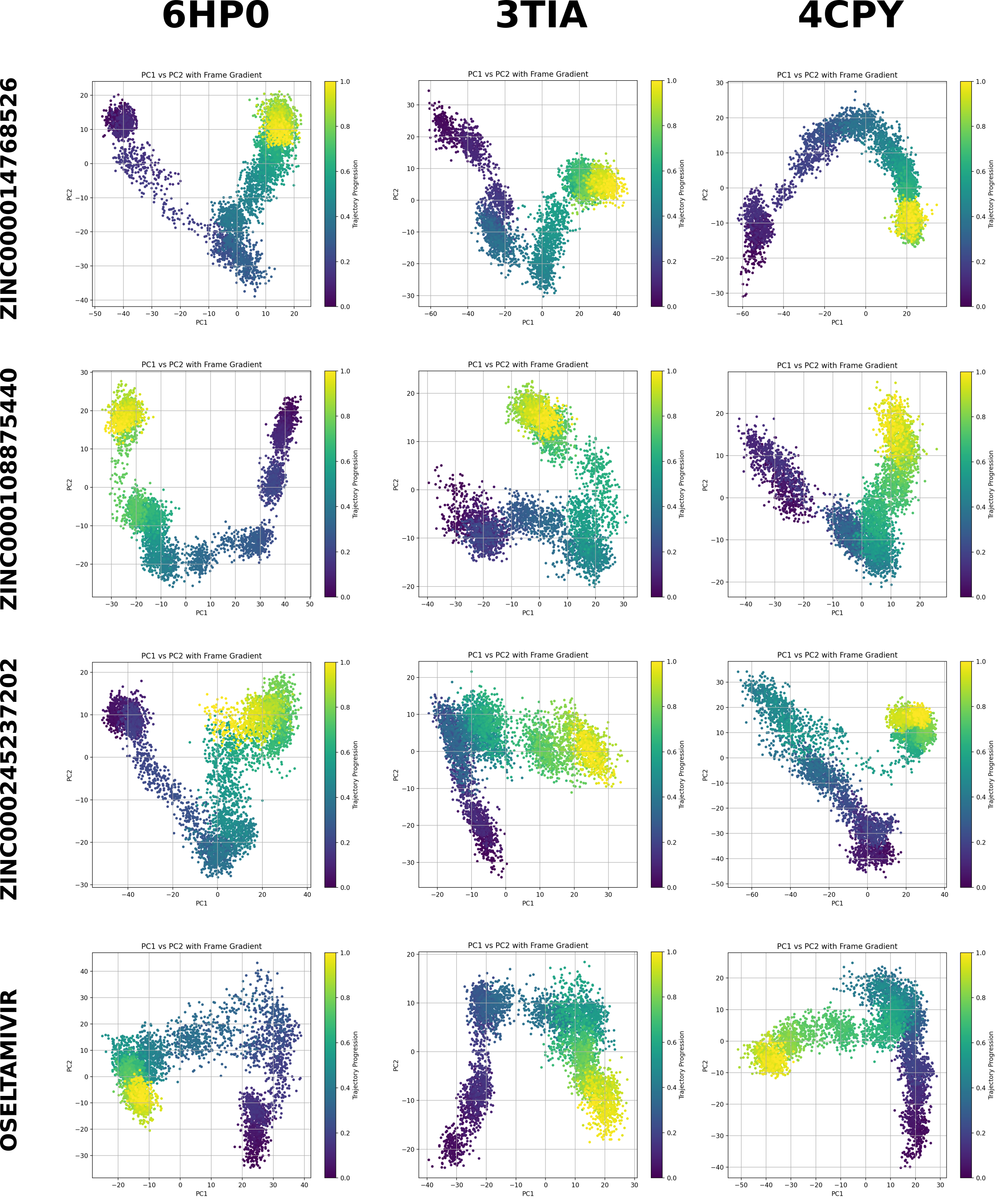

Supplement: Supplementary file 14 [file Image4.tiff]

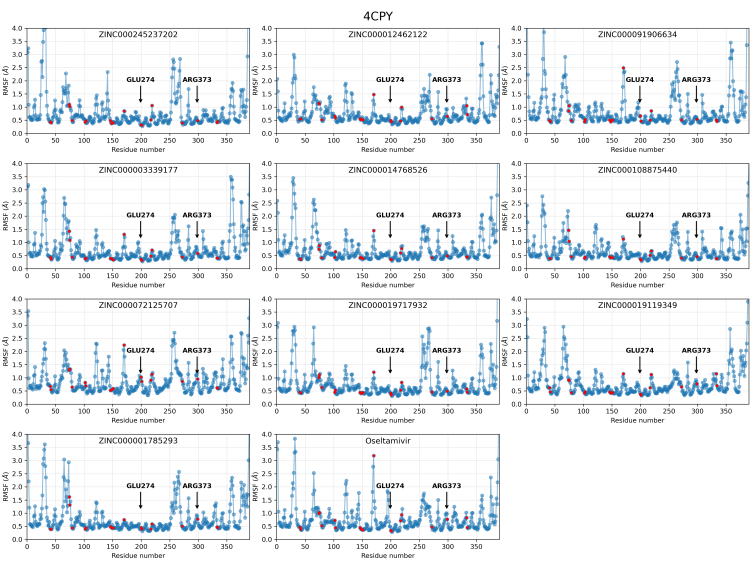

Supplement: Supplementary file 15 [file Image6.png]
